# Effects of metformin on inflammation, oxidative stress, and bone loss in a rat model of periodontitis

**DOI:** 10.1371/journal.pone.0183506

**Published:** 2017-08-28

**Authors:** Aurigena Antunes de Araújo, Aline de Sousa Barbosa Freitas Pereira, Caroline Addison Carvalho Xavier de Medeiros, Gerly Anne de Castro Brito, Renata Ferreira de Carvalho Leitão, Lorena de Souza Araújo, Paulo Marcos Matta Guedes, Sarah Hiyari, Flávia Q. Pirih, Raimundo Fernandes de Araújo Júnior

**Affiliations:** 1 Department of Biophysics and Pharmacology, Post Graduation Program Public Health / Post Graduation Program in Pharmaceutical Science, UFRN, Natal, RN, Brazil; 2 Post Graduation Program Public Health, UFRN, Natal, RN, Brazil; 3 Department of Biophysics and Pharmacology,Post Graduation Program RENORBIO,/Post Graduation Program Biological Science, UFRN, Natal, RN, Brazil; 4 Department of Morphology, Post Graduation Program in Morphology, UFC, Fortaleza, Ceará, Brazil; 5 Post Graduation Program Public Health, UFRN, Natal, RN, Brazil; 6 Department of Microbiology and Parasitology, Post Graduation Program in Parasitary Biology/Post Guaduation Biological Science, UFRN, Natal, RN, Brazil; 7 Section of Periodontics, School of Dentistry, University of California, UCLA, Los Angeles, California, United States of America; 8 Department of Morphology, Post Graduation Program in Functional and Structural Biology/ Post Graduation Program Health Science/Department of Morphology, UFRN, Natal, RN, Brazil; Charles P. Darby Children's Research Institute, UNITED STATES

## Abstract

**Aim:**

To evaluate the effects of metformin (Met) on inflammation, oxidative stress, and bone loss in a rat model of ligature-induced periodontitis.

**Materials & methods:**

Male albino Wistar rats were divided randomly into five groups of twenty-one rats each, and given the following treatments for 10 days: (1) no ligature + water, (2) ligature + water, (3) ligature + 50 mg/kg Met, (4) ligature + 100 mg/kg Met, and (5) ligature + 200 mg/kg Met. Water or Met was administered orally. Maxillae were fixed and scanned using Micro-computed Tomography (μCT) to quantitate linear and bone volume/tissue volume (BV/TV) volumetric bone loss. Histopathological characteristics were assessed through immunohistochemical staining for MMP-9, COX-2, the RANKL/RANK/OPG pathway, SOD-1, and GPx-1. Additionally, confocal microscopy was used to analyze osteocalcin fluorescence. UV-VIS analysis was used to examine the levels of malondialdehyde, glutathione, IL-1β and TNF-α from gingival tissues. Quantitative RT-PCR reaction was used to gene expression of AMPK, NF-κB (p65), and Hmgb1 from gingival tissues. Significance among groups were analysed using a one-way ANOVA. A p-value of p<0.05 indicated a significant difference.

**Results:**

Treatment with 50 mg/kg Met significantly reduced concentrations of malondialdehyde, IL-1β, and TNF-α (*p* < 0.05). Additionally, weak staining was observed for COX-2, MMP-9, RANK, RANKL, SOD-1, and GPx-1 after 50 mg/kg Met. OPG and Osteocalcin showed strong staining in the same group. Radiographically, linear measurements showed a statistically significant reduction in bone loss after 50 mg/kg Met compared to the ligature and Met 200 mg/kg groups. The same pattern was observed volumetrically in BV/TV and decreased osteoclast number (p<0.05). RT-PCR showed increased AMPK expression and decreased expression of NF-κB (p65) and HMGB1 after 50 mg/kg Met.

**Conclusions:**

Metformin, at a concentration of 50 mg/kg, decreases the inflammatory response, oxidative stress and bone loss in ligature-induced periodontitis in rats.

## Introduction

Periodontal disease is mediated by the inflammatory response in the host and bacteria in the dental tissue. In order for the disease to be established, susceptibility of the immunological response to bacterial infection is necessary [1]. The disruption of the balance between osteoblast and osteoclast activities by bacterial products and inflammatory cytokines is the main underlying cause of inflammation-induced bone loss. Complex inflammatory signals and cytokines, including Receptor Activator of Nuclear Factor kappa-B ligand (RANKL), interleukin (IL)-1β, IL-6, tumour necrosis factor-α (TNF-α), and prostaglandin E2, regulate osteoclastogenesis [2].

In addition to being a result of the changing balance in bone metabolism, periodontal disease may be affected by metabolic changes of systemic diseases, such as diabetes. Persistent hyperglycaemia leading to exaggerated immune-inflammatory responses that are induced by periodontal pathogens is likely responsible for the greater risk and severity of periodontal disease in diabetic patients [3,4].

Metformin, a biguanide, is the top choice of oral agent for the treatment of type 2 diabetes according to the guidelines (Consensus Statement of the American Diabetes Association and the European Association for the Study of Diabetes) [5]. Metformin was shown to activate the AMP-activated protein kinase (AMPK) in intact cells and *in vivo* [6]. AMPK may confer benefits in chronic inflammatory diseases independent of its ability to normalise blood glucose. Previous groups have expressed considerable interest in the identification and exploitation of its anti-inflammatory effects [7,8]. It has also been suggested that AMPK activation is responsible for the inhibition of NF-κB activation. AMPK has been shown to exert significant anti-inflammatory and immunosuppressive effects in models of inflammatory/autoimmune disease [9,10].

Metformin may stimulate osteoblast differentiation through the transactivation of genes via AMPK regulation. AMPK has been shown to increase significantly the expression of the key osteogenic genes, such as alkaline phosphatase (ALP) and osteocalcin (OC) [11]. Taken together, the aim of this study was to investigate the role of metformin in oxidative stress, inflammation and bone loss, as well as the participation of the AMPK/NF-KB (p65) and HMGB1 in periodontitis disease in a rat model.

## Materials and methods

### Animals

Two-Months-old male Wistar rats (180g) were bred and handled at the Federal University of Rio Grande do Norte (UFRN), Brazil. This study was carried out in strict accordance with the recommendations in the Guide for the Care and Use of Laboratory Animals of the National Institutes of Health. The protocol was approved by the Committee on the Ethics of Animal Experiments of the UFRN (CEUA, Permit Number: 066/2014). All surgery was performed under anesthesia, and all efforts were made to minimize suffering. In brief, animals were housed in standard conditions (12hr light/dark cycle and 22 ± 0.1°C), with standard chow and access to food and water *ad libitum*.

### Model for experimental periodontitis (EPD)

Animals were anesthetized by the IP injection of 10% ketamine (80 mg/kg, Quetamina, Vetnil, São Paulo, Brazil) and 2% xylazine (10 mg/kg, Calmium, São Paulo, Brazil). EPD was induced by the placement of a sterile nylon thread ligature (3–0; Polysuture, NP45330, São Paulo, Brazil) around the cervix of the maxillary left second molar. No ligature was added to the right contralateral side. Additionally, in 21 rats, no ligature was added to either the right or left side, which served as control animals. Animals were euthanized eleven days after the initial treatment by an i.p injection of 80 mg/kg thiopental (0.5 g Thiopentax, Cristália, São Paulo, Brazil).

### Drug treatments

Metformin hydrochloride (Met), 850mg (Medley, Campinas, São Paulo, Brazil) was dissolved in distilled water. Distilled water served as the vehicle treatment. All treatments (Met or vehicle) were administered by oral gavage 2 hours before the addition of the ligature (induction of EPD) and thereafter once daily for 10 days. The animals were assigned randomly to the following 5 groups of 21 rats each: (1) a no ligature group that received water (NL), (2) a ligature group that received water (L), (3) a ligature group treated with 50 mg/kg Met, (4) a ligature group treated with 100 mg/kg Met, and (5) a ligature group treated with 200 mg/kg Met. The dose selection for the present study was based on doses translation from animal to human studies [12,13].

### Radiographic Micro-computed tomography (microCT) measurement of ABL

At the end of the experiment (10 days after addition of the ligature and first drug treatments), animals were euthanized; maxillae were dissected and fixed in 10% buffered formalin for 24 hours and Stored in 70% alcohol. Rat maxillae were scanned using micro-computed tomography (μCT, micro-CT) (Model 1172; SkyScan, Kontich, Belgium) at 20 micrometers resolution, as previously described (Hiyari et al). Micro-CT files were converted to Digital Imaging and Communications in Medicine (DICOM) files and imported into Dolphin software for linear bone height analysis. Linear bone height analysis was performed by orienting the second molar cementoenamel junctions (CEJ) parallel to each other in the coronal plane. In the axial plane, the middle of the crown was identified and linear bone distances were recorded on the mesial aspect of the second molar on the sagittal image. Additional measurements were taken 0.3mm palatal from the middle of the crown; again recording on the mesial aspect of the second molar on the sagittal image. The linear measurements were recorded from the CEJ to the alveolar crest (AC). Each second molar received a total of 2 measurements and these values were averaged for each group. To assess volumetric bone volume/tissue volume (BV/TV) changes, samples were oriented using DataViewer (V.1.5.2 Bruker, Billerica, MA) such that the CEJ’s of the second molar were parallel to each other in the sagital and coronal planes. The axial plane showed the crowns of each molar, first, second, and third. Oriented images were analyzed using CTAn (V.1.16 Bruker, Billerica, MA). A 40 slice volume set at a threshold of 75 in the bifurcation área of the second molar was used as a region of interest for analysis. Analysis started at the slice where the second molar roots first presented with bifurcation and went down 40 slices towards the root apices. BV/TV values were recorded as a percent and averaged for each group (n≥3/group for all μCT analysis).

### Histopathological analysis and osteoclast quantification

Five maxillae per group were harvested, fixed in 10% neutral-buffered formalin, and demineralized in 5% nitric acid for 14 days. Following decalcification, specimens were dehydrated and embedded in paraffin. Four-μm thick sections were obtained in the sagittal plane. Sections were stained with hematoxylin and eosin using standard protocols[14].

Histomorphometrical evaluations were performed in the areas between the first and second molars (where the ligature had been placed). Sections were evaluated by light microscopy (40X magnification). The sectioning and subsequently the analysis by light microscopy were performed in the Laboratory of Investigation of Cancer and Inflammation (LAICI) in the Department of Morphology/UFRN.

Hematoxylin and eosin stained alveolar bone specimens (n = 5/group) were evaluated on the following parameters: inflammatory cell influx and integrity of the alveolar bone and cementum. Samples were analyzed by a histologist in a single-blind fashion and graded as follows: a score of 0 indicated that inflammatory cell infiltration was absent or sparse, was restricted to the region of the marginal gingiva, and that the alveolar process and cementum were preserved; a score of 1 indicated moderate cellular infiltration (inflammatory cellular infiltration present on the entire gingival insert), minor alveolar resorption, and intact cementum; a score of 2 indicated accentuated cellular infiltration (inflammatory cellular infiltration present in the gingiva and in the periodontal ligament), accentuated degradation of the alveolar process, and partial destruction of the cementum; and a score of 3 indicated accentuated cellular infiltration, complete resorption of the alveolar process, and severe destruction of the cementum [14]. In addition, all the osteoclasts found on the surface of the alveolar bone between the first and second molars were counted on H&E stained slides, at 20x magnification. Only osteoclasts with two or more nuclei and in contact with the bone surface were counted. The result is expressed as the average osteoclast number per experimental group, considering at least 4 animals per group. The histological and immunohistochemical analysis scores of the periodontal tissues were conducted by calibrated oral pathologists (R.F.A. Jr, A.A.A, F.Q.P and S.H).

### Immunohistochemical analysis of RANK-L, RANK, and OPG, Cathepsin, COX-2, MMP-9, SOD-1, Gpx

Thin sections of periodontal tissue (4 μm) (n = 3/group) were obtained with a microtome and were transferred to gelatin-coated slides. Each tissue section was then deparaffinized and rehydrated. The gingival and periodontal tissue slices were washed with 0.3% Triton X-100 in phosphate buffer, quenched with endogenous peroxidase (3% hydrogen peroxide), and incubated with the following primary antibodies overnight at 4°C: receptor activator of the NF-κB ligand (RANK-L), 1:400; receptor activator of NF-κB (RANK), 1:400; and osteoprotegerin (OPG), 1:400; Cathepsin, 1:400; Cycloxygenase (COX-2), 1:400; Matrix Metalloproteinase-9 (MMP-9), 1:400; Superoxide dismutase (*SOD-1*), 1:400; Glutathione Peroxidase (Gpx), 1:400 (all antibodies from Santa Cruz Biotechnology, INTERPRISE, Brazil). After primary antibody incubation, sections were washed with phosphate buffer and incubated with a streptavidin-HRP-conjugated secondary antibody (Biocare Medical, Concord, CA, USA) for 30 minutes. Immunoreactivity to RANK, RANK-L, OPG, Cathepsin, COX-2, MMP-9, SOD-1, and GPx, were visualized using a colorimetric-based detection kit following the protocol provided by the manufacturer (TrekAvidin-HRP Label + Kit from Biocare Medical, Dako, USA).

### Immunofluorescence

Three tissue sections from each animal (n = 3/group) were deparaffinized in xylene and washed in a series of concentrations of ethanol and PBS buffer. Antigen retrieval was performed by placing the sections in a 10 mM sodium citrate solution with 0.05% Tween 20 for 40 minutes at 95°C. Autofluorescence background noise was reduced by incubating the sections in 0.1% Sudan black in 70% ethanol for 20 min at room temperature (RT). The sections were incubated overnight with rabbit anti-ostecalcin primary antibody (1:100, respectively, in 1% normal goat serum; Santa Cruz Biotechnology, USA) at 4°C, washed three times in 0.2% triton X-100 for 5 min and incubated with Alexa Fluor 488- conjugated goat anti-rabbit secondary antibody (1:500 in BSA 1%) and DAPI nuclear counterstain (SIGMA, USA). Finally, the sections were mounted with Vectashield medium.

Samples were counterstained with DAPI (SIGMA, USA). Finally, the sections were mounted with Vectashield medium.Fluorescent images were obtained on a Carl Zeiss Laser Scanning Microscope (LSM 710, 20× objective, Carl Zeiss, Jena, Germany, Brazil). Known positive and negative controls were included in each batch of samples. The quantitative estimation of fluorescently-tagged osteocalcin (labeled with Alexa-fluor) was determined from digital images of at least? different areas from each section (from? specimens per group), at? x magnification, using Image J software (http://rsb.info.nih.gov/ij/). Tissue reactivity (immunofluorescence) in all groups (NL, L, and Met 50mg/kg) was assessed by computerized densitometry analysis. Contrast index measurements were obtained from the formula (selected area × 100)/total area after removal of background in regions of interest (three samples per animal).

### Malondialdehyde (MDA) levels

MDA is an end product of lipid peroxidation. To quantify the increase in free radicals in gingival samples, MDA content was measured via the assay previously described [15]. Gingival samples (Four sample for group) were suspended in Tris HCl buffer 1:5 (weight/volume) and minced with scissors for 15 sec on an ice-cold plate. The resulting suspension was homogenised for 2 min with an automatic Potter homogenizer and centrifuged at 2500 × g at 4°C for 10 min. The supernatants were assayed to determine MDA content. The absorbance of each sample was measured at 586nm. The results are expressed as nanomoles of MDA per gram of tissue.

### Glutathione (GSH) assay

GSH levels in the gingival tissues (Four sample for group) were measured as a marker for antioxidant activity. GSH content was measured via the assay described by Costa et al [16]. The gingival samples (5 samples per group) were removed and stored at −70°C in TCA (Trichloroacetic acid)and frozen in liquid nitrogen until the assay. Gingival tissue homogenate (0.25 mL of a 5% tissue solution prepared in 0.02 M EDTA) was added to 320 μL of distilled water and 80 μL of 50% TCA. The samples were then centrifuged at 3000 rpm for 15 minutes at 4°C. The supernatant (400 μL) was added to 800 μL of 0.4 M Tris buffer at pH 8.9 and 20 μL of 0.01 M 5,5-dithio-bis-(2-nitrobenzoic acid) (DTNB). The absorbance of each sample was measured at 420 nm, and the results were reported as units of GSH per milligram of tissue.

### IL-1β and TNF-α assay

Gingival sample tissues (Four sample for group) were stored at −70°C until required for each assay. The tissue collected was homogenized and processed as described by by Safieh-Garabedian et al [17]. Levels of IL-1β (detection range: 62.5–4000 pg/mL; sensitivity or lower limit of detection: 12.5 ng/mL of recombinant mouse IL-1β) and TNF-α (detection range: 62.5–4000 pg/mL; sensitivity or lower limit of detection: 50 ng/mL of recombinant mouse TNF-α) in the gingival samples (samples per group) were determined with a commercial ELISA kit (R&D Systems, Minneapolis, MN, USA), as described previously [18]. All samples were within the wavelength used in UV-VIS spectrophotometry (absorbance measured at 490 nm).

### RNA extraction and quantitative real-time RT-PCR

Total RNA from the gingival tissues (Four sample for group)was extracted using Trizol reagent (Life Technologies, California, USA) as described previously [19]. The RNA isolation and purification procedure was performed using the SV Total RNA Isolation System (Promega Corporation, USA). The RNA concentration was determined from the optical density at a wavelength of 260 nm (using an OD_260_ unit equivalent to 40 μg/ml RNA). Five micrograms of isolated total RNA were reverse transcribed to cDNA in a reaction mixture containing 4 μl 5X reaction buffer, 2 μl dNTP mixture (10 mM), 20 units of RNase inhibitor, 200 units of avian-myeloblastosis virus (AMV) reverse transcriptase, and 0.5 μg oligo(dT) primer (High-Capacity cDNA Reverse Transcription Kit, Foster City, USA) in a total volume of 20 μl. The reaction mixture was incubated at 42°C for 60 min, and the reaction was terminated by heating at 70°C for 10 min. The cDNA was stored at −80°C until further use. Gene expression was evaluated by PCR amplification using primer pairs based on published rat sequences (GADPH- Rattus norvegicus: Forward primer: AACTTGGCATCGTGGAAGG, Reverse Primer: GTGGATGCAGGGATGATGTTC; AMPK-Rattus norvegicus protein kinase, AMP-activated, alpha 2 catalytic subunit (Prkaa2), mRNA: Forward primer: AGCTCGCAGTGGCTTATCAT, Reverse Primer: GGGGCTGTCTGCTATGAGAG; NF-κB-Rattus norvegicus v-rel avian reticuloendotheliosis viral oncogene homolog A (Rela), mRNA Forward primer: TCTGCTTCCAGGTGACAGTG, Reverse Primer: ATCTTGAGCTCGGCAGTGTT; Hmgb1-Rattus norvegicus high mobility group box 1 (Hmgb1), mRNA: Forward primer: GAGTACCGCCCAAAAATCAA, Reverse Primer: TTCATCCTCCTCGTCGTCTT.

Quantitative RT-PCR was performed using Power SYBR Green master mix (Applied Biosystems, USA), and a Step One Plus thermocycler (Applied Biosystems, USA), according to the manufacturer's instructions. To the 1X PCR master mix, 2.5 μl of each cDNA was added in a final volume of 20 μl. The PCR conditions were as follows: 95°C for 5 min, 40 cycles of 30 s at 95°C, 30 s at 52–60°C (based on the target), and 60 s at 72°C. The relative quantitative fold change compared with the control (no ligature + water group) was calculated using the comparative Ct method, where Ct is the cycle number at which fluorescence first exceeds the threshold. The Ct values from each sample were obtained by subtracting the values for GADPH Ct from the target gene Ct value. The specificity of resulting PCR products was confirmed by melting curves.

### Statistical analysis

The data are presented as means ± standard error of the mean or as medians, when appropriate. One-way analysis of variance (ANOVA) followed by a Bonferroni’s test was used to compare significance among groups. A Kruskal-Wallis test followed by a Dunn’s test was used to compare medians. For radiographic linear and volumetric bone loss, data were averaged per each group and compared using a Student’s t-test. A p-value of p≤0.05 indicated a statistical significance. (GraphPad Prism 5.0 Software, La Jolla, CA, USA).

## Results

### Radiographic assessment of alveolar bone loss

**Fig 1:**
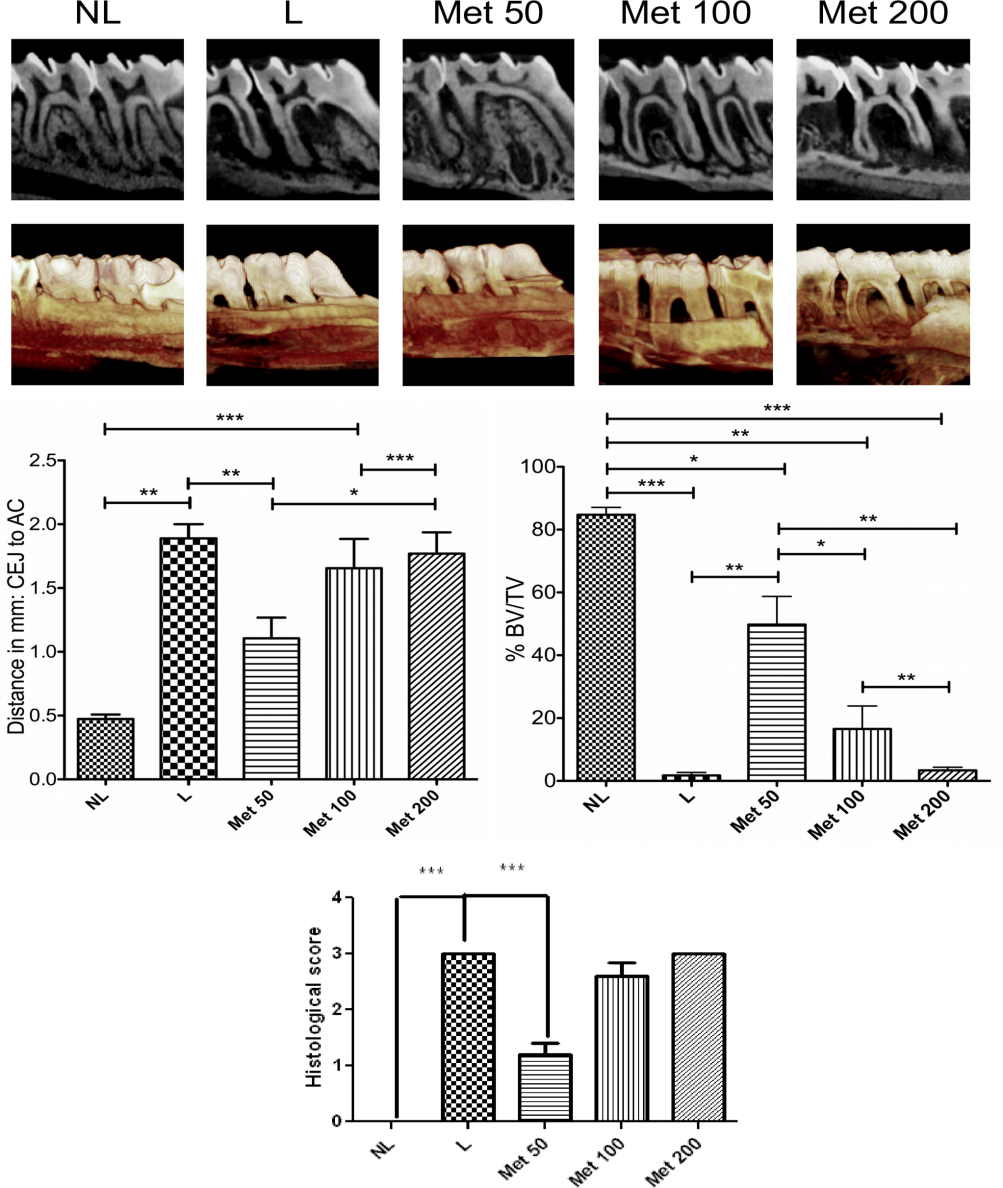
Radiographic evaluation after Met treatment and experimental periodontitis. (A) Representative sagittal 2D and 3D microCT images of no ligature (NL), ligature (L), Met 50, Met 100, and Met 200 groups. (B) Graph representing linear bone loss in the area of the mesial second molar. Data are mean ± standard area of the mean. *p<0.05, *p<0.01, ***p<0.001, (n≥3 for all groups/time points). (C) Graph representing the % BV/TV in the area under the second molar bifurcation. Data are mean ± standard area of the mean. *p<0.05, *p<0.01, ***p<0.001, (n≥3 for all groups/time points).

Rats with EPD (L), and all concentrations of Met (50, 100, and 200 mg/kg) treatment showed statistically significant more linear bone loss compared to NL (0.473mm ± 0.035mm). However, when comparing L (1.888mm ± 0.111mm) to 50 mg/kg Met (1.106 ± 0.161mm) treatment, bone loss was statistically reduced in 50 mg/kg Met treatment. All other concentrations of Met, 100 and 200 mg/kg, showed similar bone loss levels as the L group. The pattern was similar volumetrically. Focusing on the area of the bifurcation under the second molar, all groups show statistically significantly less BV/TV compared to NL (84.73% ± 2.35%). However, again, 50 mg/kg Met showed statistically more BV/TV (49.78% ± 8.96%) compared to L (1.77% ± 0.91%), 100 mg/kg Met (16.56% ± 7.28%), and 200 mg/kg Met (3.36% ± 0.98%), (Fig 1).

### Histological analysis

**Fig 2:**
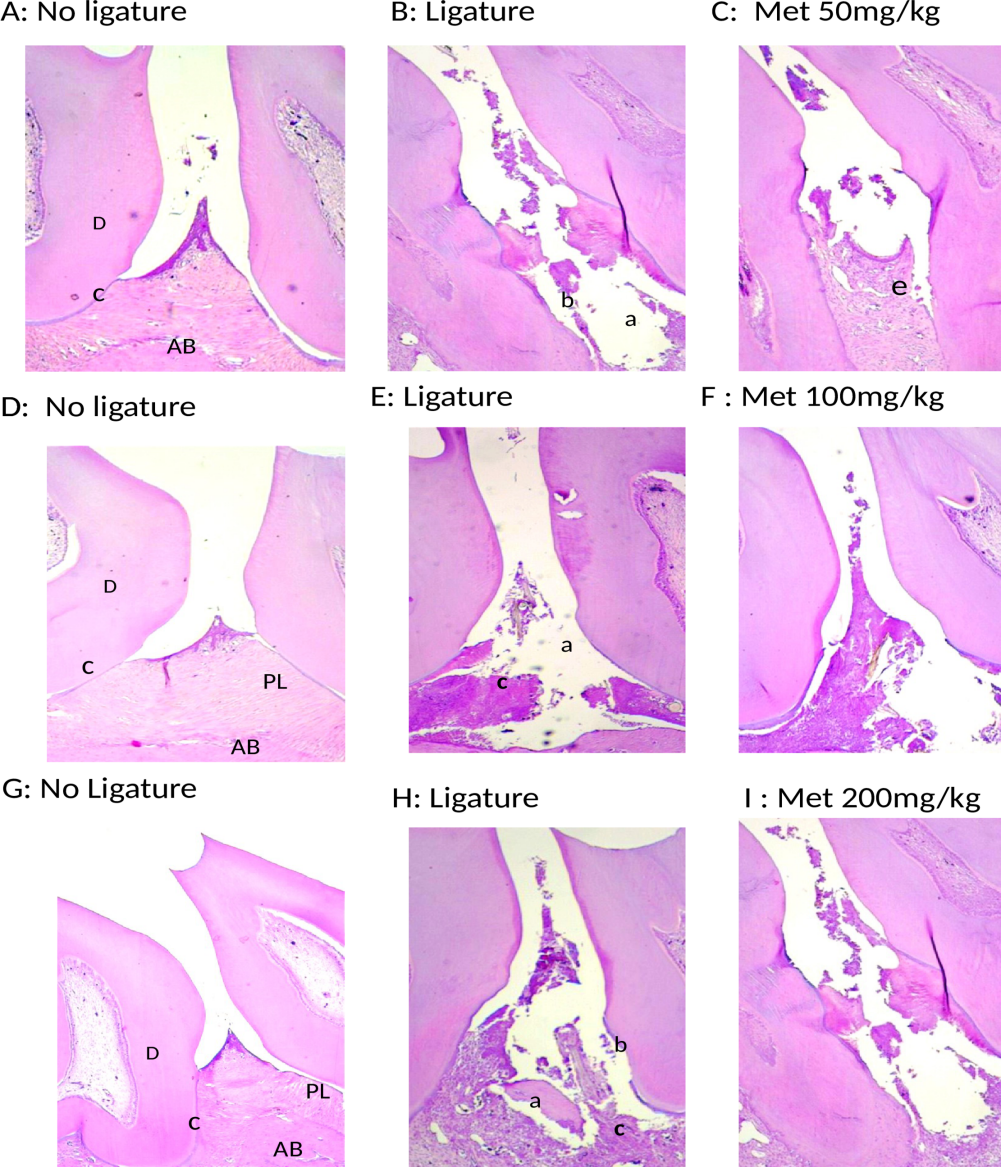
Microscopic analyses. A, D and G No ligature. B, E, H: Ligature, Periodontium from a rat with periodontitis (treated with saline) showing alveolar bone and cementum resorption (discontinuous cementum) and inflammatory cell infiltration. F: Treatment with Met (100 mg/kg) and I: Treatment with Met (200 mg/kg) showing no reduced inflammation and increased alveolar bone loss. F: Periodontium from a rat with periodontitis (treated with Met, 50 mg/kg) showing reduced inflammation and decreased alveolar bone loss. Sections were stained with H&E. Original magnification 40×. Scale bars = 100 μm. PL, periodontal ligament; D, dentin; AB, alveolar bone; C, cementum; a, bone loss; b, resorption of cementum; c, inflammatory process; e, decreased inflammation process.

Histological analysis of the region between the first and second molars of the NL group showed that the structure of the periodontium was normal and that the gingiva, periodontal ligament, alveolar bone, and cementum were easily observed (Fig 2A, 2D and 2G).

The periodontal histopathology of the animals subjected to ligature (L) that received no Met treatment revealed inflammatory cell infiltration coupled with severe destruction of the cementum and alveolar process, with animals of this group receiving a median score of 3 (Fig 2B, 2E and 2H; Fig 1).

Alveolar bone loss was reduced in the EPD-induced animals treated with 50 mg/kg Met compared with the L group that received no Met treatment (median score of 1, 1–1.5) (p<0.001) and 3 (3–3), respectively (Fig 1). This result can be clearly observed histopathologically; discrete cellular infiltration was restricted to the region of the marginal gingiva, there was preservation of alveolar bone, and intact cementum in the group treated with 50 mg/kg Met (Fig 2C).

The periodontal histopathology of the animals subjected to EPD that received treatment of 100 and 200 mg/kg Met revealed inflammatory cell infiltration coupled with severe destruction of the cementum and alveolar process, with animals of this group receiving a median score of 3 (2–3) and 3 (3–3), respectively (p>0.05) (Fig 2F and 2I; Fig 1).

**Fig 3:**
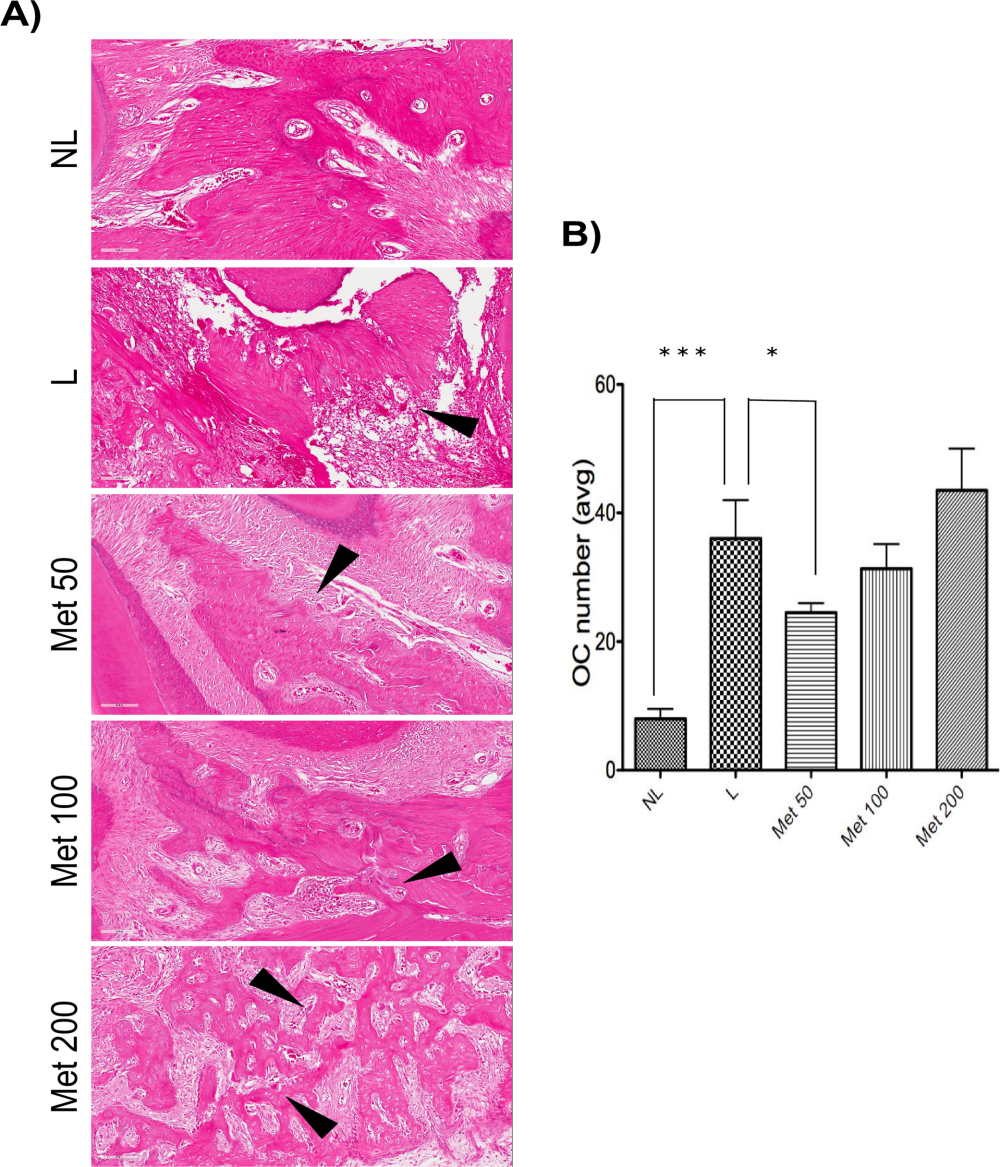
Quantitation of osteoclast numbers after MET treatment and experimental periodontitis. (A) Representative H&E stained sagittal images of no ligature (NL), ligature (L), Met 50, Met 100, and Met 200 groups. Black arrows highlight osteoclasts, multinucleated cells lining the bone areas. Sections were stained with H&E. Original magnification 20×. Noted the increased bone destruction and porosity in the ligature and Met 200 groups. (B) Graph representing osteoclast numbers quantitated (avg:average). Data are mean ± standard error of the mean. *p<0.05, ***p<0.001, (n≥2 for all groups/time points). (ANOVA test followed by Bonferrone).

The Fig 3 shows representative slides of osteoclasts (arrows) lining the alveolar bone between the first and second molars of non-ligated animals (NL) or animals subjected to EPD that received saline (L) or Met, 50, 100 or 200 mg/kg, The experimental periodontal disease significantly increased the osteoclast number. This effect was prevented by the lowest concentration of Met 50 (p<0,05) and NL (p<0.001), as illustrated in the Fig 3.

### Immunohistochemical analysis on markers of inflammation and bone loss

**Fig 4:**
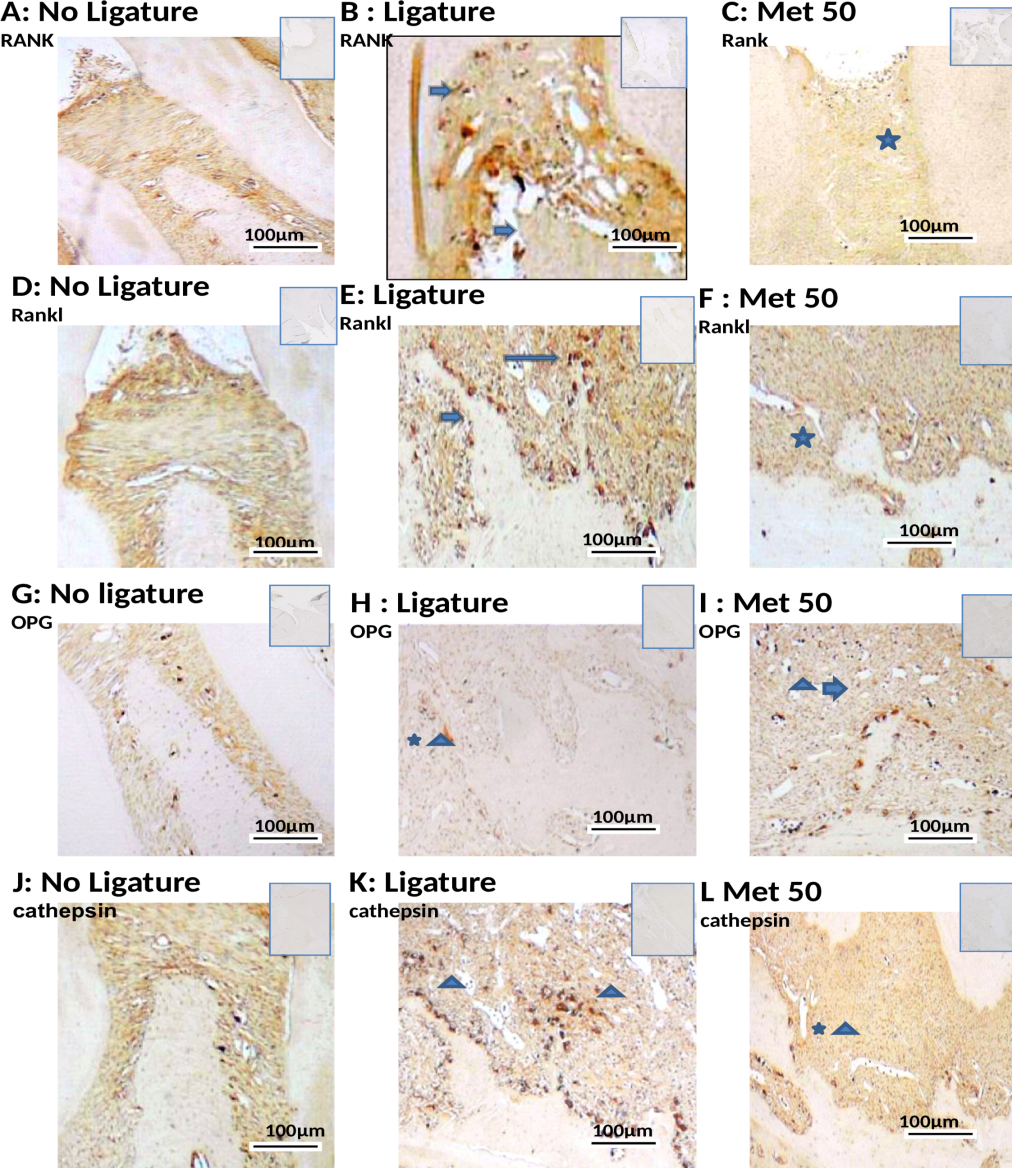
Photomicrographs of periodontal tissue of PD rats treated with Met and No ligature and Ligature groups, showing immunoreactivity to RANK, RANK-L, OPG, and Cathapsyn. Rats subjected to saline (A, D, G, J); rats subjected to ligation (B, E, H, K); rats subjected to ligation and treated with Met (50 mg/kg) (C, F, I, L). Images are shown at 40× magnification. Bar = 100 μm. Arrow indicates high or moderate labeling in the periodontal ligament or the alveolar bone. Asterisk indicates mild labeling in the periodontal ligament or the alveolar bone. Triangle and asterisk indicate mild labeling of OPG in osteoclasts. Triangle and arrow indicate high labeling of osteoclasts. Triangle indicates intense labeling of Cathepsin on alveolar bone. Asterisk and triangle indicate mild labeling of cathepsin on alveolar bone. Small picture- Upper right (non-immune or IgG controls).

**Fig 5:**
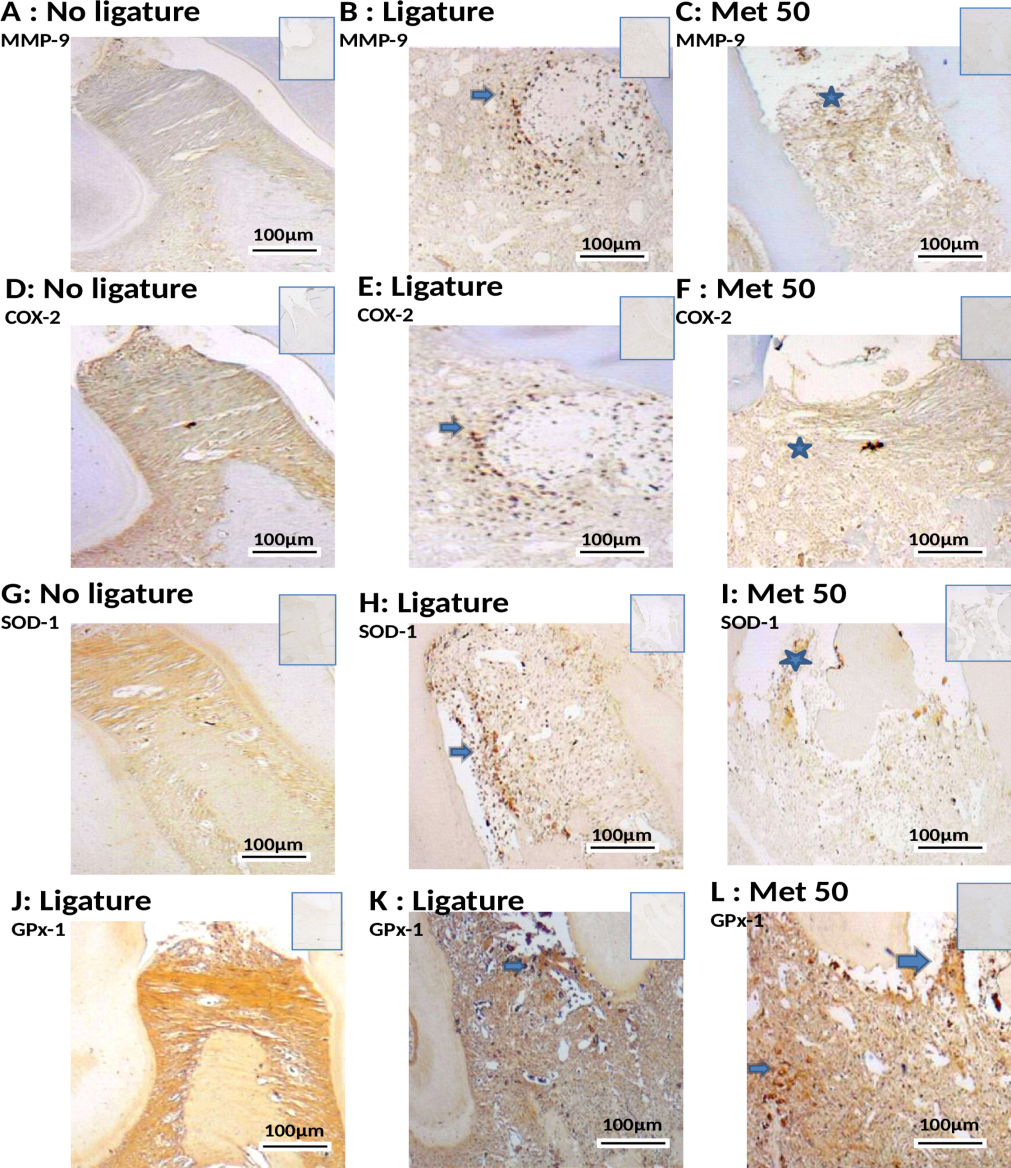
Photomicrographs of periodontal tissue of PD rats treated with Met and No ligature and Ligature groups showing immunoreactivity to MMP-9, COX-2, SOD-1 and GPX-1. Rats subjected to saline (A, D, G, J); rats subjected to ligation (B, E, H, K); rats subjected to ligation and treated with Met (50 mg/kg) (C, F, I, L). Images are shown at 40× magnification. Bar = 100 μm. Arrow indicates high or moderate labeling in the periodontal ligament or the alveolar bone. Asterisk indicates mild labeling in the periodontal ligament or the alveolar bone. Small picture- Upper right (non-immune or IgG controls)

Compared with that of the NL, the periodontium of rats subjected to EPD (L) showed marked immunostaining for the following antibodies: RANK, RANK-L, OPG, Cathepsin K, MMP-9, COX-2, SOD-1 and GPx (NL-saline, Figs 4, 5A, 5D, 5G and 5J; L-saline, Fig 5, 5B, 5E, 5H and 5K; Met 50 mg/kg, Figs 4, 5C, 5F, 5I and 5L).

Met at a concentration of 50 mg/kg, reduced the immunostaining of RANK, RANK-L, Cathepsin, COX-2, MMP-9 and SOD-1 in the periodontium of rats subjected to L (Fig 4C, 4F, 4L and Fig 5C, 5F and 5I). However, intense staining was observed for OPG and GPx in the group treated with 50 mg/kg Met (Figs 4I and 5L, respectively). The L group showed high immunostaining of RANK, RANK-L, Cathepsin, COX-2, MMP-9, SOD-1 and GPx in the periodontium of rats subjected to EPD (Fig 4B, 4E, 4K and Fig 5B, 5H and 5K). Mild staining was observed for OPG, in the L group (Fig 4H).

### Confocal immunofluorescence

**Fig 6:**
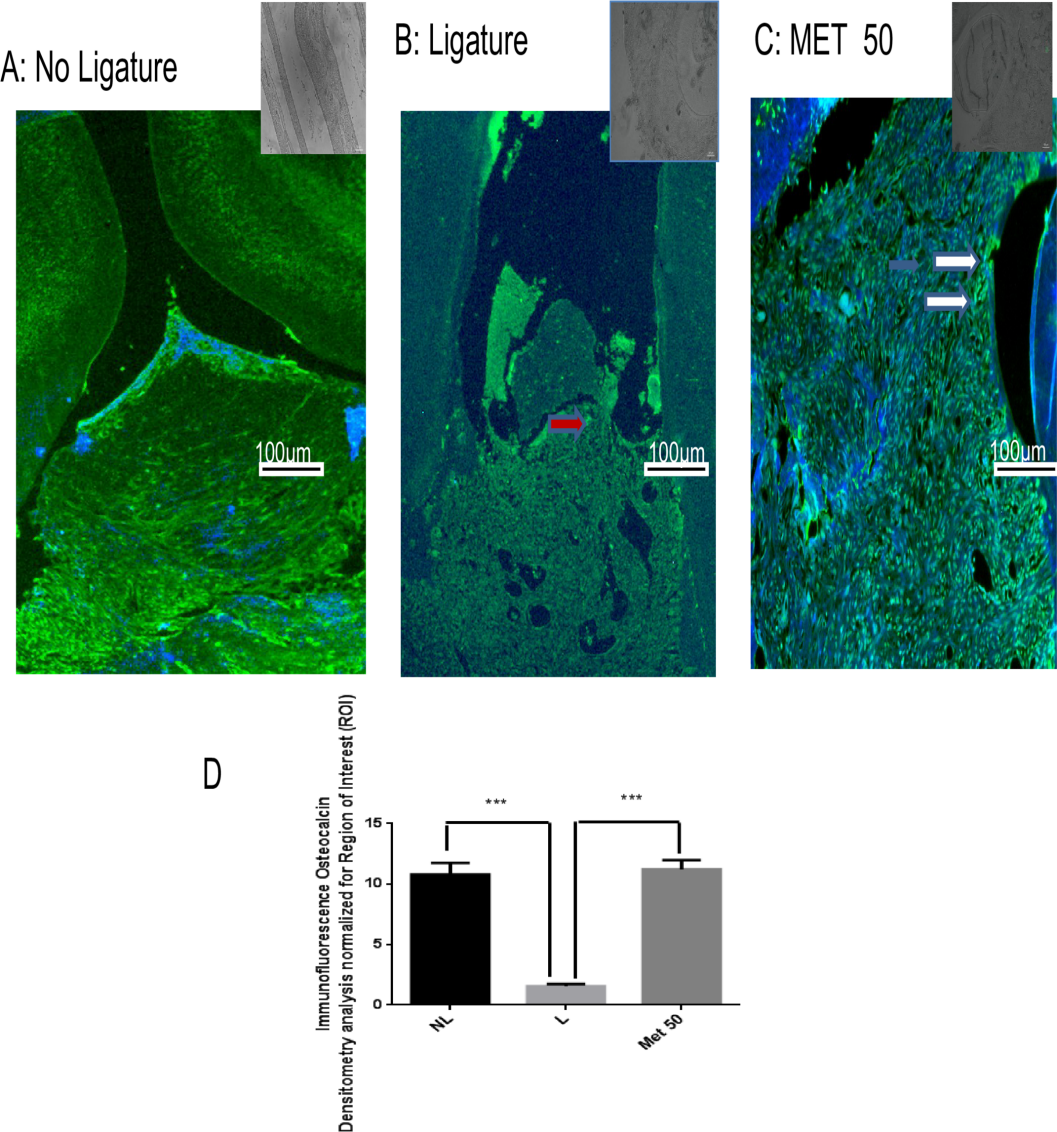
Representative confocal photomicrographs of fluorescently-tagged osteocalcin (green) in the periodontal tissue. Samples were counterstained with DAPI nuclear counterstained (blue). (A) No ligature group: The rat periodontium presents osteocalcin labelling, showing presence of osteoblasts. (B) Weak osteocalcin labelling (red arrows) was seen in the ligature group. (C) Osteocalcin labelling (white narrow) was diffuse and strong in the Met50 group. Scale bar, 100 mm, 10x. (G,H). (D) Densitometric analysis confirmed significant increases in osteocalcin immunoreactivity in the Met (50mg/kg) that were blocked in the positive control group. Five immunofluorescence sections from each animal in each group were analyzed (N = 3 animals per group) (***p < 0.001, Kruskal-Wallis test followed by Dunn’s test). Small picture- Upper right (non-immune or IgG controls)

Cellular osteocalcin labeling (green) was strong and diffuse in the Met 50 mg/kg group (Fig 6), weak in the L group (Fig 6), and moderate in the NL group (Fig 6). Densitometric analysis confirmed that there were significantly increased osteocalcin immunoreactivities in the Met 50mg/kg group (p<0.001), relative to the L group (Fig 6). Densitometric analysis confirmed that there were significantly increased osteocalcin immunoreactivities in the Met 50 mg/kg group (p<0.001), relative to the positive control group (Fig 6).

### Effect of MET treatment on markers of oxidative stress

**Fig 7:**
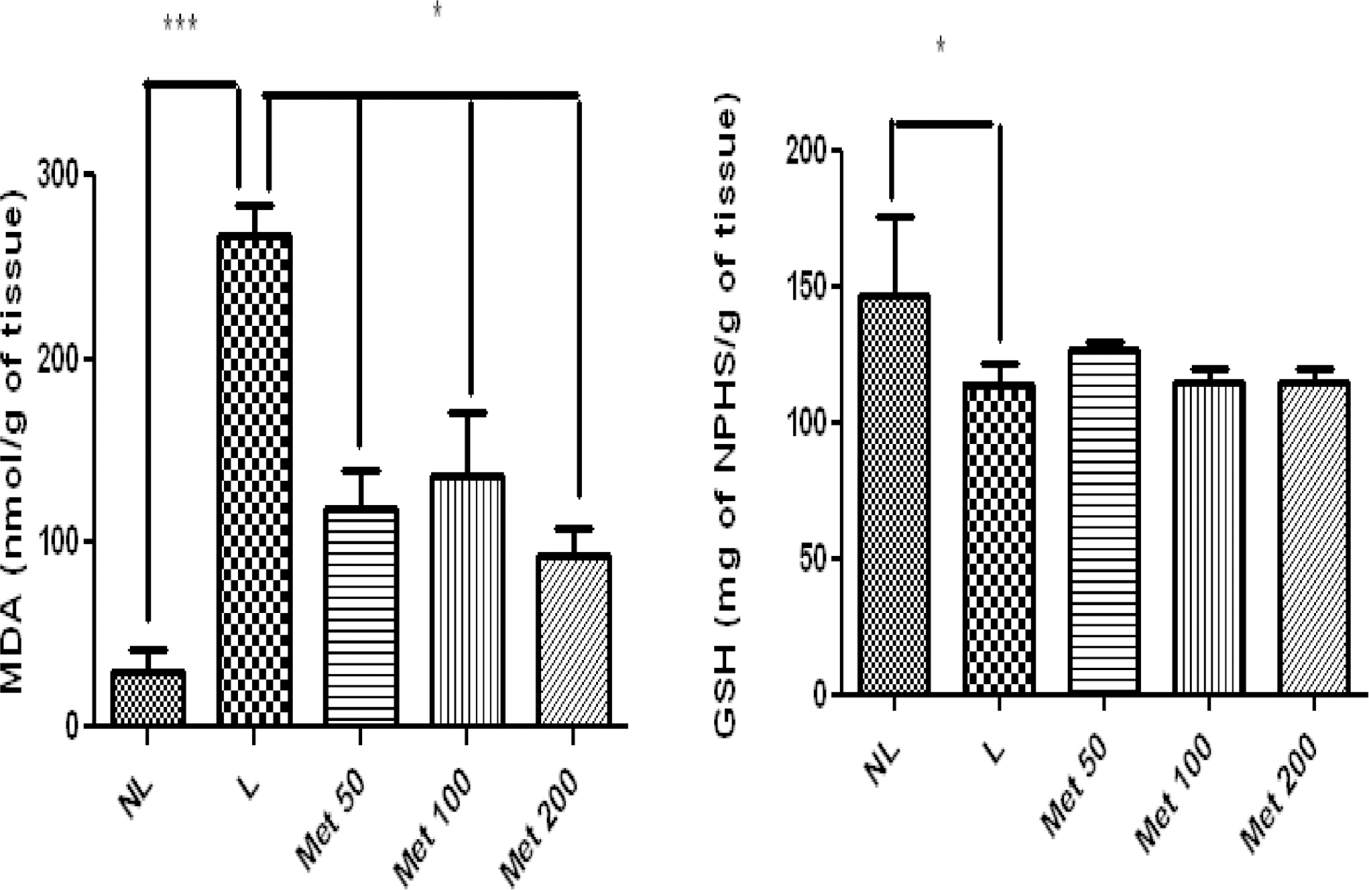
MDA and GSH in NL, L, and groups treated with 50 mg/kg, 100 mg/kg, and 200 mg/kg Met (*p <0.05, **p < 0.01). (ANOVA test followed by Bonferrone).

The NL group showed significantly lower levels of MDA compared to the L-saline group (p<0.001). Treating EPD-induced rats with all doses of Met reduced MDA levels compared to the L group (p<0.05). The treatment of EPD-induced rats with Met did not significantly increase levels of GSH (p>0.05) compared with those of the L group (Fig 7). However, GSH was significantly reduced in the L group (p>0.05) (Fig 7).

### Effect of MET treatment on inflammatory activity

**Fig 8:**
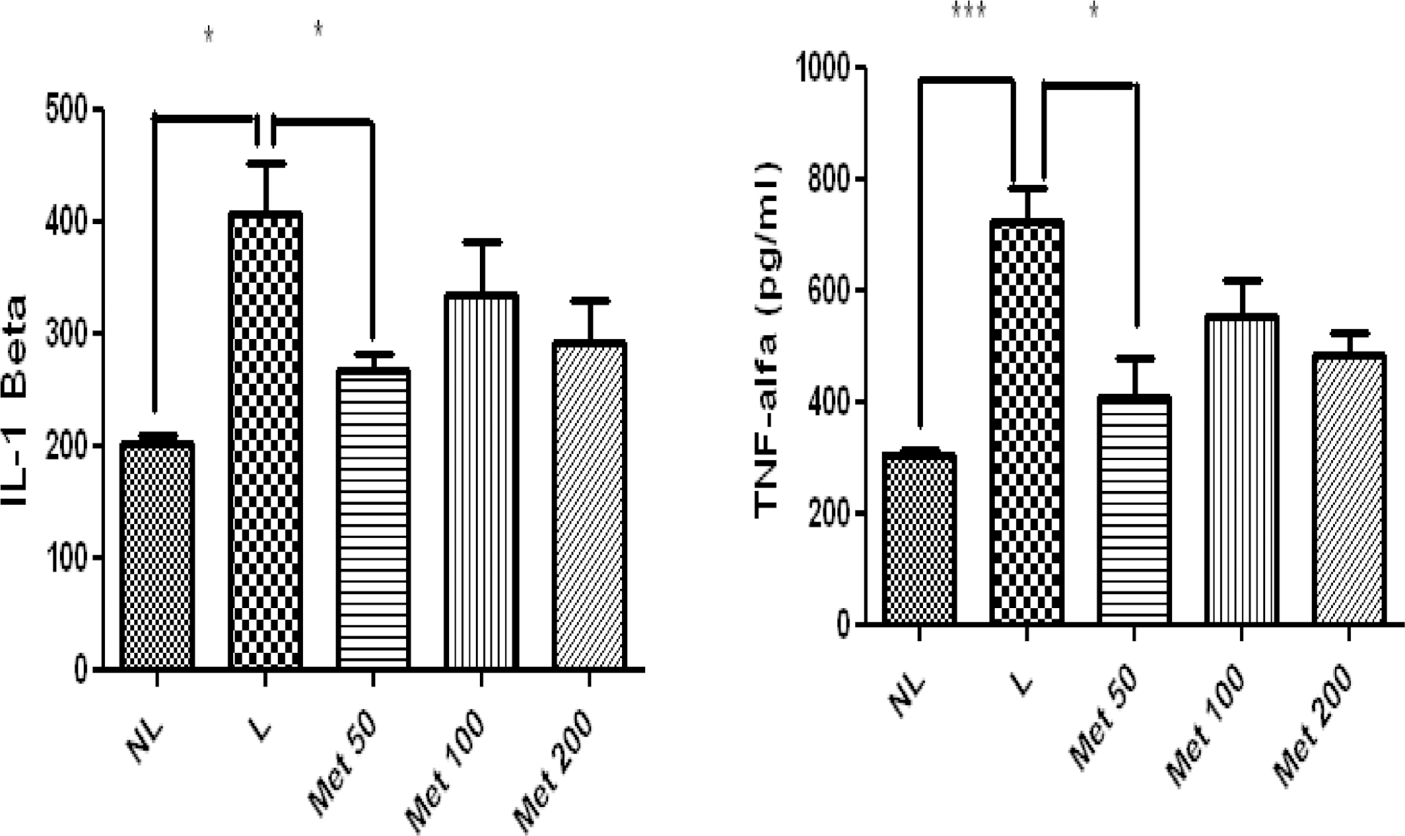
Levels of A) IL-1β, B) TNF- α, in NL, L, and Met-treated animals (50 mg/kg, 100 mg/kg, and 200 mg/kg) (*p <0.05). (ANOVA test followed by Bonferrone).

The levels of the proinflammatory cytokine IL-1β were significantly decreased in the group treated with 50 mg/kg Met compared to the L group (p<0.05). Additionally comparing, the NL and L group, the NL group showed significantly less IL-1β (p<0.05). Furthermore, TNF-α levels were significantly reduced in the 50 mg/kg Met group (p<0.05). Moreover, comparing the NL and L group, the L group had significantly more TNF-α levels (Fig 8).

**Fig 9:**
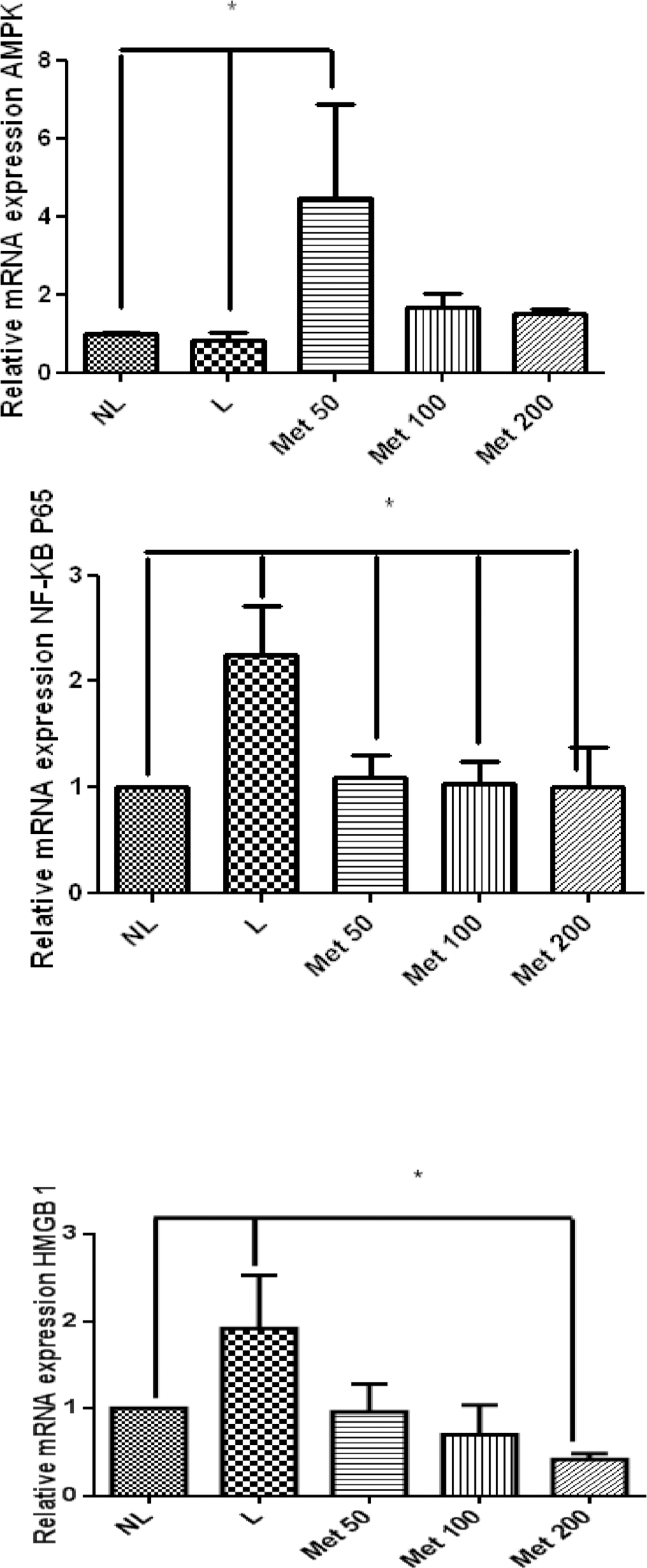
Real-Time RT-PCR analysis of gene expression. Met effects on AMPK, NF-KB p65, and Hmgb1 mRNA expression in rats with Periodontal disease. The expression of AMPK mRNA was increased in Met 50 mg/kg group (*p< 0.01, Fig 8) and decreased in L and NL group. The expression of NF-Kb p65 mRNA was decreased in All Met groups and NL group compared to the L group (*p< 0.05)Hmgb1 mRNA levels appeared to be lower in the Met 50 mg/kg group and NL group compared to the L group (*p>0.05, Fig 8). (N = 5 animals per group; ANOVA test followed by Bonferrone).

All Met doses in the groups with induced EPD reduced the expression of NF-κB p65 (*p*<0.01) and the expression of high mobility group box 1 (Hmgb1) (p<0.05) compared to those of the L-saline group (Fig 9). The 50 mg/kg Met treatment increased the expression of AMPK compared with the L and NL group (*p*<0.05), but 100 and 200 mg/kg Met did not significantly increase expression (Fig 9).

## Discussion

Periodontal disease is an immune-inflammatory disease characterize by connective tissue breakdown, loss of attachment, and alveolar bone resorption [20]. Evidence has linked reactive oxygen species (ROS) to the pathological destruction of connective tissue in periodontal disease [21,22]. The hydroxyl radical can initiate a classical chain reaction known as lipid peroxidation, leading to bone reabsorption [23]. Hydrogen peroxide stimulates phosphorylation of the Factor Nuclear kappa B-inhibitor of kappa B **(**NF-κB–IκB) complex, activating NF-κB and facilitating nuclear translocation and downstream movement of proinflammatory cytokines, such as Tumour Necrosis Factor-α (TNF-α), which are very important in the pathogenesis of periodontal disease [24]. Superoxide generated at the osteoclast–bone interface is involved in bone matrix degradation [25]. Superoxide is removed from tissue by superoxide dismutase (SOD). NF-κB is a transcription factor that is specifically activated when T cells are exposed to oxidant stress [26].

In the present study, all Met doses caused significant reductions in the formation of malondialdehyde, a major end product of lipid peroxidation. In the ligature group treated with 50 mg/kg Met, the low degree of histological staining for enzymes involved in the neutralisation of ROS, such as SOD, implies a productive antioxidant effect, in which the formation of oxidative stress products is reduced by the stimulation of antioxidant enzyme activity. These results are corroborated by data reported by Akalin et al (ANO) [27], who found greater SOD activity in inflamed gingiva from patients with chronic periodontitis than in healthy gingiva from controls. SOD activity has been shown to increase directly after the occurrence of oxidative stress [28]. One of the oxidative stress reduction outcomes for the metformin-treated groups can be seen in the reduction of the gene expression of NF KB. Asehnoune,et al (ANO) showed that ROS can modulate NF-B-dependent transcription [29].

Although NFKB expression was low in all metformin-treated groups at day 10, phenotypic characteristics of an active inflammatory process with high levels of IL-1β and TNF-alpha were still found in the groups treated with 100mg/ kg and 200mg /kg metformin. These findings may contribute to the lack of effect of the higher concentrations of Met in preventing bone loss. Accordingly, the lowest dose (50mg / kg) of metformin resulted in a significant reduction in IL-1β and TNF-alpha levels, as well as COX-2 immunostaining. A transcription factor directly related to the mechanism of action of metformin is activated protein kinase (AMPK). AMPK provides a target capable of mediating the beneficial metabolic effects of metformin. AMPK is a key for metabolic enzymes such as 3-hidroxi-3-metilglutaril CoA redutase (HMGCoA redutase) and acetil CoA carboxilase (ACC) [30]

Other important role of AMPK is the regulation of bone metabolism [31],. Osteocalcin (OC) is a bone-specific protein, specifically synthesized in osteoblasts. OC is recognized as one of the markers of the mature osteoblast phenotype [32]. Kasai et al evaluated the effects of metformin on matrix mineralization by osteoblasts. For both MC3T3-E1 and primary osteoblasts, Matrix mineralization induced by ascorbic acid and β-glycerophosphate were significantly inhibited by the presence of 2mM metformin. Lower concentrations of metformin failed to affect matrix mineralization in both cell types, but high concentrations of metformin affected matrix mineralization. The degree of AMPKα phosphorylation was well correlated with the inhibition of matrix mineralization in metformin-stimulated osteoblasts [33]. On the other hand, findings indicate the participation of AMPK by negatively regulating the RANKL[34]. Lee at al found that RANKL itself can positively stimulate AMPK activation up to the 50ng/ml concentration of RANKL, which implies that stimulated AMPK will inhibit RANKL, however concentrations of 100ng/ml RANKL were not able to activate AMPK[34], indicating a possible saturation of this negative feedback.

In our study we were able to verify in vivo that low doses of metformin (50mg/kg) reduced bone loss and increased relative mRNA expression AMPK, with osteocalcin elevation and reduced RANKL immunolabeling, thereby indicating an increase in the presence of mature osteoblasts and a reduction of osteoclasts number. On the other hand, the higher doses of metformin (100mg/kg or 200mg/kg) appear to have negatively regulated AMPK. This finding may partially explain why the higher doses failed to reduce the bone loss induced by periodontitis, considering some studies showing that activation of AMPK promotes bone formation in vitro[35].

HMGB1, a nuclear protein released from activated macrophages or injured cells, displays pro-inflammatory cytokine-like properties once it enters the extracellular space and HMGB1-induced osteoclastogenesis [36]. Once in the extracellular space, HMGB1 seems to be an important participant in RANKL-induced osteoclastogenesis *in vitro* and *in vivo* [37]. In our study it was possible to verify a reduction of the gene expression of HMGB1 in the group treated with the dose of 50mg / kg metformin and consequently lower immunolabeling of RANKL, which supports our findings of a lower osteoclast activity in this group, and consequently lower bone loss.

Another important finding is the disorganization of the extracellular matrix in the periodontal tissue. Selective production of MMP-9 can lead to the acceleration of matrix degradation in pathological conditions, such as periodontitis [38]. In this study, weak labelling for MMP-9 was observed in the 50-mg/kg MET group.

Our data showed that the best bone loss results were found when metformin was administered at a lower dose of 50 mg / kg, correlating drug dosage from animal to human dose**[13],** We verified that the therapeutic dose of metformin in humans occurs in a range of 1700mg-3000mg/day. The dose of 50 mg/kg used in this study is below the therapeutic dose (approximately 567 mg/day), while the groups treated with 100 or 200 mg/kg is equivalent to the therapeutic dose used in diabetic humans. However it is important to consider that the animals in this study were not diabetic, since our objective was to verify the pleiotropic effect of metformin in periodontal disease. Thus, we believe that this effect of low dose metformin in animals with periodontal disease without diabetes interfered differently in bone loss. However, we also believe that although doses of 100mg/kg or 200mg/kg are equivalent to the human therapeutic dose, when administered in animals without diabetes it can affect matrix mineralization by osteoblasts, as seen in the in vitro study carried out by Kasai et al. (ANO) [33].

Our study demonstrated the ability of Met to regulate AMPK, NF-κB p65, and Hmgb1 gene expression, resulting in the reduction of malondialdehyde, TNF-α, and IL-1β levels and control bone loss. Ultimately, Metformin showed anti-inflammatory, antioxidant activity and decreased bone loss.

## References

[1] Cekici A, Kantarci A, Hasturk H, Van Dyke TE (2014) Inflammatory and immune pathways in the pathogenesis of periodontal disease. Periodontol 2000 64: 57–80. 10.1111/prd.12002 PMC4500791 24320956

[2] Henderson B, Nair SP, Ward JM, Wilson M (2003) Molecular pathogenicity of the oral opportunistic pathogen Actinobacillus actinomycetemcomitans. Annu Rev Microbiol 57: 29–55. doi: 10.1146/annurev.micro.57.030502.090908 14527274 10.1146/annurev.micro.57.030502.090908

[3] Al-Khabbaz AK (2014) Type 2 diabetes mellitus and periodontal disease severity. Oral Health Prev Dent 12: 77–82. doi: 10.3290/j.ohpd.a31223 24619786 10.3290/j.ohpd.a31223

[4] Demmer RT, Holtfreter B, Desvarieux M, Jacobs DR Jr., Kerner W, Nauck M, et al (2012) The influence of type 1 and type 2 diabetes on periodontal disease progression: prospective results from the Study of Health in Pomerania (SHIP). Diabetes Care 35: 2036–2042. doi: 10.2337/dc11-2453 22855731 10.2337/dc11-2453 PMC3447825

[5] Woo V (2009) Medical management of hyperglycemia in type 2 diabetes: a consensus algorithm for the initiation and adjustment of therapy: a consensus statement of the American Diabetes Association and the European Association for the Study of Diabetes: response to Nathan et al. Diabetes Care 32: e34; author reply e37-38. doi: 10.2337/dc08-2093 19246585 10.2337/dc08-2093

[6] Hawley SA, Gadalla AE, Olsen GS, Hardie DG (2002) The antidiabetic drug metformin activates the AMP-activated protein kinase cascade via an adenine nucleotide-independent mechanism. Diabetes 51: 2420–2425. 12145153 10.2337/diabetes.51.8.2420

[7] Salt IP, Palmer TM (2012) Exploiting the anti-inflammatory effects of AMP-activated protein kinase activation. Expert Opin Investig Drugs 21: 1155–1167. doi: 10.1517/13543784.2012.696609 22694351 10.1517/13543784.2012.696609

[8] Hattori Y, Suzuki K, Hattori S, Kasai K (2006) Metformin inhibits cytokine-induced nuclear factor kappaB activation via AMP-activated protein kinase activation in vascular endothelial cells. Hypertension 47: 1183–1188. doi: 10.1161/01.HYP.0000221429.94591.72 16636195 10.1161/01.HYP.0000221429.94591.72

[9] Guma M, Wang Y, Viollet B, Liu-Bryan R (2015) AMPK Activation by A-769662 Controls IL-6 Expression in Inflammatory Arthritis. PLoS One 10: e0140452 doi: 10.1371/journal.pone.0140452 26474486 10.1371/journal.pone.0140452 PMC4608670

[10] Mayer ML, Blohmke CJ, Falsafi R, Fjell CD, Madera L, Turvey SE, et al (2013) Rescue of dysfunctional autophagy attenuates hyperinflammatory responses from cystic fibrosis cells. J Immunol 190: 1227–1238. doi: 10.4049/jimmunol.1201404 23264659 10.4049/jimmunol.1201404

[11] Jang WG, Kim EJ, Bae IH, Lee KN, Kim YD, Kim DK, et al (2011) Metformin induces osteoblast differentiation via orphan nuclear receptor SHP-mediated transactivation of Runx2. Bone 48: 885–893. doi: 10.1016/j.bone.2010.12.003 21147283 10.1016/j.bone.2010.12.003

[12] Reagan-Shaw S, Nihal M, Ahmad N (2008) Dose translation from animal to human studies revisited. Faseb Journal 22: 659–661. doi: 10.1096/fj.07-9574LSF 17942826 10.1096/fj.07-9574LSF

[13] Reagan-Shaw S, Nihal M, Ahmad N (2008) Dose translation from animal to human studies revisited. FASEB J 22: 659–661. doi: 10.1096/fj.07-9574LSF 17942826 10.1096/fj.07-9574LSF

[14] Leitao RF, Ribeiro RA, Chaves HV, Rocha FA, Lima V, Brito GA (2005) Nitric oxide synthase inhibition prevents alveolar bone resorption in experimental periodontitis in rats. J Periodontol 76: 956–963. doi: 10.1902/jop.2005.76.6.956 15948691 10.1902/jop.2005.76.6.956

[15] Siddique YH, Ara G, Afzal M (2012) Estimation of lipid peroxidation induced by hydrogen peroxide in cultured human lymphocytes. Dose Response 10: 1–10. doi: 10.2203/dose-response.10-002.Siddique 22423225 10.2203/dose-response.10-002.Siddique PMC3299524

[16] Costa CMd, Santos RCCd, Lima ES (2006) A simple automated procedure for thiol measurement in human serum samples. Jornal Brasileiro de Patologia e Medicina Laboratorial 42: 345–350.

[17] Safieh-Garabedian B, Poole S, Allchorne A, Winter J, Woolf CJ (1995) Contribution of interleukin-1 beta to the inflammation-induced increase in nerve growth factor levels and inflammatory hyperalgesia. Br J Pharmacol 115: 1265–1275. 7582555 10.1111/j.1476-5381.1995.tb15035.x PMC1908795

[18] Kendall C, Ionescu-Matiu I, Dreesman GR (1983) Utilization of the biotin/avidin system to amplify the sensitivity of the enzyme-linked immunosorbent assay (ELISA). J Immunol Methods 56: 329–339. 6833765 10.1016/s0022-1759(83)80022-2

[19] Chomczynski P, Sacchi N (2006) The single-step method of RNA isolation by acid guanidinium thiocyanate-phenol-chloroform extraction: twenty-something years on. Nature Protocols 1: 581–585. doi: 10.1038/nprot.2006.83 17406285 10.1038/nprot.2006.83

[20] Biju T, Shabeer MM, Amitha R, Rajendra BP, Suchetha K (2014) Comparative evaluation of serum superoxide dismutase and glutathione levels in periodontally diseased patients: an interventional study. Indian J Dent Res 25: 613–616. doi: 10.4103/0970-9290.147105 25511061 10.4103/0970-9290.147105

[21] Kantarci A, Oyaizu K, Van Dyke TE (2003) Neutrophil-mediated tissue injury in periodontal disease pathogenesis: findings from localized aggressive periodontitis. J Periodontol 74: 66–75. doi: 10.1902/jop.2003.74.1.66 12593599 10.1902/jop.2003.74.1.66

[22] Katsuragi H, Ohtake M, Kurasawa I, Saito K (2003) Intracellular production and extracellular release of oxygen radicals by PMNs and oxidative stress on PMNs during phagocytosis of periodontopathic bacteria. Odontology 91: 13–18. doi: 10.1007/s10266-003-0022-1 14505184 10.1007/s10266-003-0022-1

[23] Emerit J, Michelson AM (1982) [Free radicals in medicine and biology]. Sem Hop 58: 2670–2675. 6297065

[24] Chapple IL (1996) Role of free radicals and antioxidants in the pathogenesis of the inflammatory periodontal diseases. Clin Mol Pathol 49: M247–255. 16696085 10.1136/mp.49.5.m247 PMC408069

[25] Key LL Jr., Wolf WC, Gundberg CM, Ries WL (1994) Superoxide and bone resorption. Bone 15: 431–436. 7917583 10.1016/8756-3282(94)90821-4

[26] Schreck R, Rieber P, Baeuerle PA (1991) Reactive Oxygen Intermediates as Apparently Widely Used Messengers in the Activation of the Nf-Kappa-B Transcription Factor and Hiv-1. Embo Journal 10: 2247–2258. 2065663 10.1002/j.1460-2075.1991.tb07761.x PMC452914

[27] Akalin FA, Toklu E, Renda N (2005) Analysis of superoxide dismutase activity levels in gingiva and gingival crevicular fluid in patients with chronic periodontitis and periodontally healthy controls. J Clin Periodontol 32: 238–243. doi: 10.1111/j.1600-051X.2005.00669.x 15766365 10.1111/j.1600-051X.2005.00669.x

[28] Redl H, Gasser H, Schlag G, Marzi I (1993) Involvement of oxygen radicals in shock related cell injury. Br Med Bull 49: 556–565. 8221022 10.1093/oxfordjournals.bmb.a072630

[29] Asehnoune K, Strassheim D, Mitra S, Kim JY, Abraham E (2004) Involvement of reactive oxygen species in Toll-like receptor 4-dependent activation of NF-kappa B. J Immunol 172: 2522–2529. 14764725 10.4049/jimmunol.172.4.2522

[30] Hardie DG, Carling D (1997) The AMP-activated protein kinase—fuel gauge of the mammalian cell? Eur J Biochem 246: 259–273. 9208914 10.1111/j.1432-1033.1997.00259.x

[31] Tokuda H, Kato K, Natsume H, Kondo A, Kuroyanagi G, Matsushima-Nishiwaki R, et al (2012) Involvement of AMP-activated protein kinase in thrombin-stimulated interleukin 6 synthesis in osteoblasts. J Mol Endocrinol 49: 47–55. doi: 10.1530/JME-11-0165 22645249 10.1530/JME-11-0165

[32] Kondo A, Otsuka T, Kato K, Matsushima-Nishiwaki R, Kuroyanagi G, Mizutani J, et al (2013) AMP-activated protein kinase regulates thyroid hormone-stimulated osteocalcin synthesis in osteoblasts. Int J Mol Med 31: 1457–1462. doi: 10.3892/ijmm.2013.1349 23588610 10.3892/ijmm.2013.1349

[33] Kasai T, Bandow K, Suzuki H, Chiba N, Kakimoto K, Ohnishi T, et al (2009) Osteoblast differentiation is functionally associated with decreased AMP kinase activity. Journal of Cellular Physiology 221: 740–749. doi: 10.1002/jcp.21917 19725053 10.1002/jcp.21917

[34] Lee YS, Kim YS, Lee SY, Kim GH, Kim BJ, Lee SH, et al (2010) AMP kinase acts as a negative regulator of RANKL in the differentiation of osteoclasts. Bone 47: 926–937. doi: 10.1016/j.bone.2010.08.001 20696287 10.1016/j.bone.2010.08.001

[35] Shah M, Kola B, Bataveljic A, Arnett TR, Viollet B, Saxon L, et al (2010) AMP-activated protein kinase (AMPK) activation regulates in vitro bone formation and bone mass. Bone 47: 309–319. doi: 10.1016/j.bone.2010.04.596 20399918 10.1016/j.bone.2010.04.596 PMC3629687

[36] Zhou Z, Han JY, Xi CX, Xie JX, Feng X, Wang CY, et al (2008) HMGB1 regulates RANKL-induced osteoclastogenesis in a manner dependent on RAGE. Journal of Bone and Mineral Research 23: 1084–1096. doi: 10.1359/jbmr.080234 18302500 10.1359/JBMR.080234 PMC2679382

[37] Zhou Z, Han JY, Xi CX, Xie JX, Feng X, Wang CY, et al (2008) HMGB1 regulates RANKL-induced osteoclastogenesis in a manner dependent on RAGE. J Bone Miner Res 23: 1084–1096. doi: 10.1359/jbmr.080234 18302500 10.1359/JBMR.080234 PMC2679382

[38] Esfahanian N, Shakiba Y, Nikbin B, Soraya H, Maleki-Dizaji N, Ghazi-Khansari M, et al (2012) Effect of metformin on the proliferation, migration, and MMP-2 and -9 expression of human umbilical vein endothelial cells. Mol Med Rep 5: 1068–1074. doi: 10.3892/mmr.2012.753 22246099 10.3892/mmr.2012.753 PMC3493092

